# To Implement or Not to Implement? A Commentary on the Pitfalls of Judging the Value and Risks of Personalized Prognostic Statistical Models

**DOI:** 10.2196/69341

**Published:** 2025-05-19

**Authors:** Linda Baumbach, Walter Hötzendorfer, Jan Baumbach

**Affiliations:** 1 Center for Bioinformatics, Universität Hamburg, Hamburg, Germany; 2 Department of Health Economics and Health Services Research, University Medical Center Hamburg-Eppendorf, Martinistrasse 52, Hamburg, Germany, 49 407410590; 3 Research Institute – Digital Human Rights Center, Vienna, Austria

**Keywords:** prognostic models, ethics, clinical relevance, Benefit-Risk score, survey-based models

## Abstract

Prognostic models in medicine have garnered significant attention, with established guidelines governing their development. However, there remains a lack of clarity regarding the appropriate circumstances for (1) creating and (2) implementing tools based on models with limited performance. This commentary addresses this gap by analyzing the pros and cons of tool development and providing a structured outline that includes critical questions to consider in the decision-making process, based on an example of patients with osteoarthritis. We propose three general justifications for the implementation of survey-based models: (1) mitigation of expectation bias among patients and clinicians, (2) advancement of personalized medicine, and (3) enhancement of existing predictive information sources. Nevertheless, it is crucial to acknowledge that implementing such models is always context-dependent and may harm certain patients, necessitating careful consideration of the withdrawal of tool development and implementation in specific cases. To facilitate the identification of these scenarios, we delineate 16 possibilities following the implementation of a personalized prognostic model and compare the consequences to a current one-size-fits-all treatment recommendation at a population level. Our analysis encompasses the possible patient benefits and harms resulting from implementing or not implementing personalized prognostic models and summarizes them. These findings, together with context-related factors, are important to consider when deciding if, how, and for whom a personalized prognostic tool should be created and implemented. We present a checklist of questions and an Excel sheet calculation table, allowing researchers to weigh the benefits and harms of creating and implementing a personalized prognostic model at a population level against one-size-fits-all standard care in a structured and standardized manner. We condense this into a single value using a uniform Benefit-Risk Score formula. Together with context-related factors, the calculation table and formula are designed to aid researchers in their decision-making process on providing a personalized prognostic tool and deciding for or against its complete or partial implementation. This work serves as a foundation for further discourse and refinement of tool development decisions for prognostic models in health care.

## Background

The digital transformation of health care necessitates harvesting insights from massive datasets by exploiting the potential of modern artificial intelligence and machine learning solutions and allowing, among others, the prediction of health outcomes for one specific treatment based on specific patient characteristics. Their medical application has attracted increasing attention and focus on the predictive power of the emerging complex statistical models in medicine [1]. Research to develop such prognostic models includes nine stages: (1) defining a relevant clinical outcome and how to measure it, (2) selecting the predictor variables, (3) choosing the dataset, (4) developing the predictive model, (5) conducting internal and external validation and testing, (6) presenting and interpreting the model predictions, (7) licensing the predictive model, (8) maintaining the predictive model, and (9) performing ongoing evaluation of the impact of the predictive model [2]. Their potential impact could also be evaluated after external validation and before implementation [3]. However, while research findings independent of their outcome (here, model performance) should always be published and made available to the public, the literature lacks a discussion on performance requirements for converting personalized prognostic models with weak performances into tools and implementing them. This might be due to their context-dependent nature; however, there is still a need for more guidance on universal factors to consider in the implementation decision step regarding designing a tool since there are also commonalities across different health conditions and scenarios, particularly at the population level.

In the following sections, we outline and discuss a decision-making process for deciding whether or not to further design and implement a weakly performing personalized prognostic model tool based on an example in our field of expertise—osteoarthritis.

## Recent Example and Research Question

Recently, we presented the validation of a prognostic outcome model for patients with knee osteoarthritis [4]. The initial treatment recommendation for all these patients is education and exercise therapy; unfortunately, this treatment is not always implemented [5-7]. Our study aims to inform clinicians and patients on the personalized expected outcomes following the initial treatments, motivating them to follow evidence-based treatment recommendations and to identify patients who can only expect minor pain relief—for whom, therefore, skipping the recommended first-line treatment could be considered. So far, the average expected improvement values in pain intensity (of about 14 points on a visual analog scale [VAS] of 0=no pain to 100=worst pain) from before to after participating in patient education and exercise therapy could be used to motivate patients and clinicians. Respecting patient characteristics, our personalized models predict about 7% of the outcomes more correctly than utilizing the average improvement value if allowing a deviation of ±15 VAS points. The average “predictor” has a fail rate of 49%, while our personalized predictor has a lower fail rate of 42%. The price to pay for the 7% performance improvement with the personalized model is having the patient answer 11 questions via a web-based questionnaire (~2‐3 min).

Similar accuracy rates were reported in a previous attempt with a smaller cohort of approximately half the size despite the incorporation of more potential predictor variables. Hence, no better personalized predictive models can be expected to be trainable from currently existing data [8].

Now, we face the dilemma of deciding whether this somewhat limited predictability of the personalized prognostic model, though better than utilizing the available average “predictor” of expected changes in pain, should be made publicly available in a tool and further evaluated and implemented in health care or not. Similar limitations have been reported for several predictive models in other health care areas, for example, in the study by Silva et al [9].

## Three Arguments for Implementing a Survey-Based Outcome Prediction Model

A general argument in favor of designing and implementing a prognostic tool based on a personalized prognostic model, including information on the uncertainties around the predictions, is that patients, as well as clinicians, overestimate the effects of tests and treatments [10,11]. Thus, a prognostic tool that informs not only on the expected outcome but also the uncertainties regarding the outcome and the correctness of the prediction, fosters truly informed shared decision-making between patients and their clinicians. This holds true for any outcome prediction tool.

A second argument in favor of evaluating the impact of a personalized model implementation based on survey data is that personalized outcome predictions align well with the omnipresent precision medicine trend. This trend promises personalized, holistic biopsychosocial health care using data-driven decision support systems that utilize high-tech computational tools like artificial intelligence. It is essential to distinguish between precision and personalized care, as outlined by Delpierre et al [12]. In precision medicine, prognostic models focus on biological factors based on “hard” wet-lab data [12]. This trend moves health care backward toward a biomedical model [12]. Therefore, providing prognostic models that incorporate survey data covering psychological and social factors is essential to support holistic, personalized care, though, given the relatively “soft” robustness of the data, these models often have limited performance compared to molecular wet-lab data, as illuminated in our osteoarthritis example above [9].

Our third argument for designing, evaluating, and implementing a personalized prognostic tool into real-world practice is true for any personalized model utilization that is compared to standard one-size-fits-all care, which is recommended based on average outcome values. The predictive power of our personalized model being higher than population-based predictors relying on subpopulation averages is an indication for implementation. However, if the overall predictability using personalized models is still limited, as in our case, providing personalized outcome predictions to patients might have an unfavorable impact on the placebo effect (ie, positive expectations toward the outcome improve the outcome) and nocebo effect (ie, negative expectations toward the outcome worsen the outcome) compared to providing generic predictive and average values. It is known that tailored printed information (to an individual) has a more significant impact on behavior change than targeted (to a population group) or generic (to the general population) printed information [13]. Thus, the placebo effect and the nocebo effect will likely increase if personalized expectations on the treatment outcome are communicated to a patient. However, the degree to which they will increase remains uncertain. Nonetheless, consequently, personalized or tailored prognostic models probably need to perform better than generic or average outcome predictions. The key question is now what is considerable? A statistical significance of *P*<.05? A minimal effect strength? With respect to the above example, is a robust but small improvement of 7% considerable? Although it is crucial to resolve this dilemma, to the best of our knowledge, there is no evidence on the different effects of true-positive, true-negative, false-positive, and false-negative information provided to patients by either personalized, generic, or average-based predictions, which is challenging for the decision process.

## Investigating Potential Outcomes

**Figure 1.**
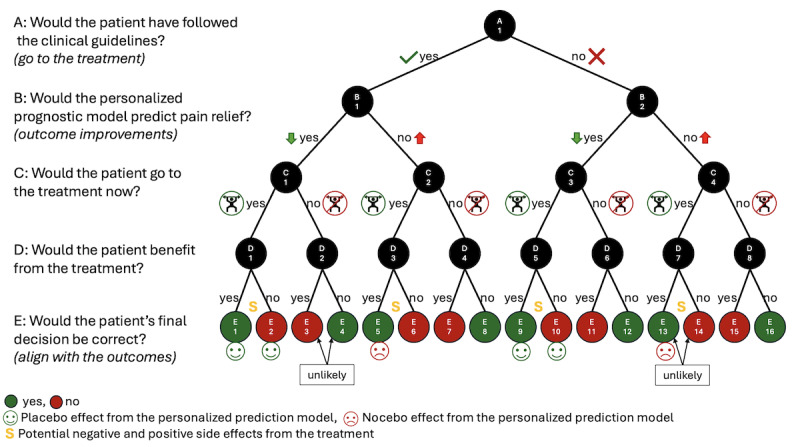
Possible outcomes for patients utilizing our personalized prognostic model depending on their behavior following treatment recommendations of the personalized prognostic model*.*

To move forward in the decision-making process of whether to create a tool suitable for clinical practice, we focus on the 16 possibilities that could result from introducing a personalized prognostic model (Figure 1). In evaluating these possibilities, which depend on the patient’s behavior and the outcome predictions, we focus on the ethical foundation of medical professionalism to do no harm and have converted our numeric outcome into a binary to ease the discussion. In Figure 1, all 16 possibilities are indicated at the bottom, highlighting if the patient’s decision aligned with the outcome.

The current clinical guidelines recommend patient education and exercise therapy for all patients; thus, in theory, all patients receive these treatments as indicated in the possibilities below node B1 in Figure 1. However, in reality, some patients do not follow the recommendation of treatment (possibilities below node B2), of which some could be motivated for the treatment through our personalized prognostic model (possibilities below node D5). These are the individuals on whom a personalized prognostic tool could have the highest positive impact. The possibilities illustrated by the subtrees below nodes D2 and D7 are unlikely, as they represent patients who contradict their initial treatment decisions despite aligned recommendations from the personalized prediction tools. For instance, a patient initially inclined toward treatment might unexpectedly decline after a favorable prognosis from the prediction tool. In the possibilities below nodes D6 and D8, using the prognostic tool does not lead to any differences in the outcomes. In the subtree possibilities of node D1 and in E5, the personalized prognostic model would only have a minor influence on the outcome through a placebo or nocebo effect. While in possibilities below node D1 the placebo effect might be strengthened by the personalized prognostic tool independent of the outcome, in node E5, patients who would utilize the prognostic model could be affected by a nocebo effect and therefore experience less improvement as compared to when they would not have used the prognostic tool. The subtrees below nodes D4 and D5 are the most interesting ones and include the most severely affected individuals (node E7); they are essential to consider when analyzing whether the (negative) impact on the individuals is within an acceptable range. Depending on the outcome below node D4, the patients could be harmed by or benefit from the personalized prognostic tool. If the patient would have participated in the treatment but did not do so due to a personalized outcome prediction of increasing pain, this could be beneficial to them if they would not benefit from the treatment. However, it could also be harmful if the patient would have benefited and does not experience pain relief due to their decision to not participate in the treatment because of a false-negative personalized outcome prediction (node E7). In the subtree below node D5, the evaluation of the personalized prognostic tool depends again on the outcome. These patients would not have participated in the treatment without utilizing the personalized prognostic tool. Thus, if a patient is motivated by the personalized outcome prediction of decreased pain, this patient may experience the highest positive impact of the personalized prognostic model by pain relief, which was not given before (node E9). If the patient does not benefit from the treatment (node E10), they might still profit from a placebo effect. In deciding whether to create a prognostic tool or not, the “benefits” and “harms” of these 16 possibilities need to be weighed against each other at a population level, and whether the “harm” experienced by the most severely “harmed” group is acceptable at all needs to assessed. Additionally, potential positive and negative side effects of the treatment on secondary outcomes (ie, the subtrees of nodes D1, D3, D5, and D7) should be considered in the decision-making process.

## The Benefit-Risk Score to Capture the Benefits and Harms at a Population Level

First, the number of patients who would benefit from or be harmed by utilizing the prognostic model ($B_{i}$) needs to be investigated for each of the 16 above-outlined possibilities with $n_{i}$ patients in each of the 16 (ie, i=1...16) groups. This number $B_{i}$ can be positive (benefit) or negative (harm). Similarly, the number of patients who would experience positive or negative side effects (${SE}_{i}$) as well as placebo or nocebo effects (${PNE}_{i}$) must be estimated for each possibility. Again, these numbers can be positive or negative. The 3 numbers must be on the same scale and make sense relative to each other in the real world, that is, if the benefit is 3 times higher than the placebo effect, the corresponding $B_{i}$ should be 3 times higher than the respective ${PNE}_{i}$. The implementation of the Benefit-Risk Score (*BRS*) for the population across all 16 groups may then be calculated using Equation 1 below. Positive values (ie, BRS>0) indicate that a personalized prediction model should be implemented, while negative values indicate a higher potential for harm and thus suggest refraining from implementing the model.


(1) $$BRS=\sum_{i=1}^{16}n_{i}(B_{i}+PNE_{i}+SE_{i})$$

To simplify the application of the formula and to enter estimated numbers, we implemented the decision tree of Figure 1 as well as Equation 1 into an easy-to-use Excel calculation sheet provided as Multimedia Appendix 1, and filled it with example data on osteoarthritis. We assume that there are a total of 1000 patients, of which one half would follow the recommended treatment and the other half would not. If all patients utilize the personalized prediction model instead, we assume that the model would predict outcome improvements for three-fourths of the patients. We then assume that 29/30 of the patients would follow a positive prediction of the personalized model if they also wanted to participate in the treatment a priori and follow a negative prediction if they did not want to participate in the treatment a priori. Further, since motivating patients to actively do something (treatment) might be harder than convincing them to do nothing (no treatment), a positive prediction would change the mind of only one half of those who did not want to participate in the treatment a priori and a negative prediction would not change the mind of one-third of those who wanted to participate in the treatment a priori. Finally, we assume that three-fifths of the model predictions would be correct regarding the benefit of the treatment [4,8]. In the next step, we evaluate if, on average, a patient per group would experience benefits, placebo or nocebo effects, and side effects to a larger, the same, or a lower extent compared to when they would not have utilized the personalized prediction model. We quantify the benefit effects with 3, 0, and −3. However, if the benefit relies solely on prevention or wasting time, we give 1 and −1 points, respectively. For the placebo and side effects, we give 1, 0, or −1 points. Finally, in our example, summarizing and multiplying the quantified effects with the number of patients reveals 832 positive effect-points, which needs to be divided by the assumed 1000 patients, leading to a number >0. Hence, at a population level, it would be recommended to create and implement the tool, though some patients (n=40) would be harmed, as they would no longer be receiving a treatment they would actually benefit from. In doubt, one could provide only clinicians with access to the tool, allowing them to reduce the negative impact by using it only with patients who are hesitant to participate in the recommended treatment, and if this data may be saved, the model may be improved over time.

However, giving only clinicians access to the tool might lead them to decide for the patient instead of together with the patient (joint decision-making), opening another ethical dimension for discussion, as ultimately, it would be the patient, not the clinician, suffering most from a suboptimal or even harmful treatment.

## Limitations of Weighting at a Population Level

As our example illustrates, there are several assumptions taken, which are subjective estimates and unlikely to be true. One of these assumptions is that there is no correlation between the a priori decision of the patient to participate in the treatment and a positive prediction by the model. Another important assumption relates to the weighing of the benefits, placebo and nocebo effects, and side effects. These numbers are based on estimates only, as there is no literature available on the effect sizes for our given example. The interplay and combination of estimated case numbers with estimated benefit values lead to some limitations of the formula. The Excel calculation sheet allows some experimentation with these values and highlights the effects (eg, when we assume that the side effect leads to +3 or −3 points, the outcome would be 1026 effect-points).

For other examples, judging the side effects may be even more challenging. If one rare side effect leads to lifelong disability, but most of the patients experience health benefits, would the side effects be positive or negative? This would be difficult to integrate into rating the average side effect. From an ethical point of view, there might thus be harms or side effects like death, which carry so much weight that benefits and positive side effects on the other side cannot outweigh them.

Furthermore, it should be noted that in our example, we binarized our numeric outcome, which is another simplification. Also, our consideration solely respects one clinical outcome and ignores other health outcomes and economic implications. Particularly, the benefits of another health outcome, which in our formula could be considered side effects, could be significant to a patient in the decision process. In our osteoarthritis example, quality of life or mobility could be alternative outcomes to be respected and included in the equation.

A final limitation of our formula and model is that it only allows the comparison of introducing a personalized prediction model over a current one-size-fits-all treatment suggestion. It does not consider any alternative treatment options. Thus, the probability of any alternative treatment leading to a “better” outcome remains uncertain. Such differential, multitask prognostic models, often incorporated in shared decision-making tools, would be more comprehensive but harder to discuss. Our simple model and running example are sufficient to illustrate our case and, more particularly, evaluate if a personalized prognostic model should be converted into a tool or not.

## Context-Related Factors

Finally, it should be noted that it is important to consider context-related factors prior to a tool’s development and implementation.

If the outcome of the Excel calculation is small or negative, an easy way to reduce the harm and, particularly, the nocebo effect of a model would be to offer it only to patients who initially refuse to participate in the first-line treatment. To secure such a partial implementation, however, clinicians would need to be involved as gatekeepers, which is against the trend of patients and clinicians meeting at eye level.

Further, prior to any implementation, potential barriers and facilitators for the usage of a tool should be considered. These factors can be at the tool or intervention level, the professional or person level, the organizational level, or in the external context [14]. Examples could be poor design, limited trust in technical tools, lack of equipment to use the tool, and lack of legal constraints, respectively.

Furthermore, when implementing prognostic model tools, it is important to consider which alternative treatment options are available and what the expected outcomes would be for those. To allow for a fair comparison and also to include patients’ preferences, shared decision-making tools are developed, which often include the findings from 2 or more prognostic models.

These are, however, beyond the scope of this paper, as is a detailed list of all potential barriers and facilitators to the usage of the tool.

## How to Use and Apply the Formula With Examples

In consequence, since the formula is an oversimplification, and since most of the time, there will be a lack of data and validated quantification, it is unrealistic to apply the formula in real-life scientific practice. Instead, it is meant as a mathematical illustration of a perfect utilitarian gold standard. It is just a visualization of an ideal, allowing the medical research community to stick to a guideline that can be followed. To allow an estimation for the formula, the supplementary Excel calculation sheet may be used. However, more importantly, due to the several assumptions that need to be taken to apply the formula, we recommend utilizing the checklist in Textbox 1 in advance. The checklist contains the questions whose answers are required to fill out the calculation sheet as well as additional potential influencing factors of implementation. Based on the results of the checklist, one should consider using the calculation sheet and implementing the tool or instead begin further investigations (eg, regarding validated numbers for the assumptions or on barriers and facilitators for an implementation). Finally, in answering these questions, we strongly recommend the incorporation of potential users of the tool—clinicians and patients.

Textbox 1. **A checklist to determine if worst- or best-case scenario calculations and implementation should be further considered.**

1. How many patients could benefit from the personalized outcome prediction (by motivating them for or against a treatment that benefits or harms them)?
2. How many patients could be harmed by the personalized outcome prediction (by motivating them for or against a treatment that harms or benefits them)?
3. How many patients would experience positive side effects, and how many would experience negative side effects?
4. How would the placebo or nocebo effect influence the outcomes?
5. On a uniform scale, how much would the benefits, harms, nocebo or placebo effects, and side effects weigh?
6. Are there additional health outcomes to be considered (maybe as side effects)?
7. Can the harm for some patients be mediated by a partial implementation (only for clinicians)?
8. Which barriers and facilitators exist at the tool or intervention level?
9. Which barriers and facilitators exist at the personal level?
10. Which barriers and facilitators exist at the institutional level?
11. Which barriers and facilitators exist in the external context?

## Generalizability

The discussed possibilities to consider when evaluating the creation of personalized prognostic tools based on weak performances on one treatment hold for all kinds of personalized outcome prediction models and tools beyond our running example in osteoarthritis. Particularly, the checklist and supplementary Excel calculation sheet may be applied to other treatments and conditions, including a one-size-fits-all approach. However, it should be noted that our example case is, in a way, nontypical, as the intervention investigated (ie, education and exercise therapy) has minimal negative side effects—only muscle soreness—and potentially positive side effects on general health, as increased physical activity benefits almost everyone. Therefore, the present example emphasizes the potential benefits and placebo and nocebo effects and only moderately touches on the additional aspects of negative side effects, which weigh more for other treatments and typically ease the decision-making process. For our example, even though estimated, there is still limited information on the occurrence of the outlined possibilities and weight of the outcomes. Conducting a respective study would be resource-demanding, ultimately leading us to the following question: is a practical clinical evaluation necessary despite the overall limited performance of our model, or would that be a waste of resources that also puts the trial participants who receive a false personalized prediction at risk?

## Conclusion

This paper addresses the challenges of deciding to develop a personalized prediction tool based on a weak-performing model or not. The decision of in which cases in particular a weak-performing personalized prognostic tool should be implemented and how is beyond the scope of this publication and needs further investigation, though we provide some general thoughts.

The general hope is that over time, with more and deeper data being fed back for model improvement, the personalization performance of prognostic tools as decision support systems will increase because of implementation. However, in general, and as discussed, deciding when a preliminary model is good enough to be converted into a tool to allow model improvements over time is challenging. One opportunity would be to include a clinician as a gatekeeper and safety guard. Consequently, one would provide such a weak-performing personalized prognostic tool only to clinicians and not directly to patients, so that the clinician can intervene and individually decide to use the tool and to share the information gained from its potential utilization.

In conclusion, we provide a checklist and corresponding Excel calculation sheet, which may help researchers to decide whether or not to create a prognostic model tool either for patients and clinicians or for clinicians alone. If a tool is only made available to clinicians due to its limited performance, it should be regularly updated with new information to eventually reach a performance level good enough to allow patients to utilize it without the harm outweighing the benefit.

## Supplementary material

10.2196/69341 Multimedia Appendix 1 Risk-benefit calculation table.

## Abbreviations

VAS: visual analog scale
